# Genome Sequence of a Copper-Resistant Strain of *Acidovorax citrulli* Causing Bacterial Fruit Blotch of Melons

**DOI:** 10.1128/genomeA.00310-15

**Published:** 2015-04-23

**Authors:** Tielin Wang, Yuwen Yang, Tingchang Zhao

**Affiliations:** State Key Laboratory for Biology of Plant Diseases and Insect Pests, Institute of Plant Protection, Chinese Academy of Agricultural Sciences, Beijing, China

## Abstract

Bacterial fruit blotch (BFB) of melons is a seed-borne disease caused by *Acidovorax citrulli*. We determined the draft genome of *A. citrulli* Tw6. The strain was isolated from a watermelon collected from Beijing, China. The *A. citrulli* Tw6 genome contains 5,080,614 bp and has a G+C content of 68.7 mol%.

## GENOME ANNOUNCEMENT

Bacterial fruit blotch (BFB) of melons leads to important economic losses to cucurbits worldwide (1–3). This seed-borne disease is caused by the Gram-negative bacterium *Acidovorax citrulli*. The management of BFB would involve the use of resistant cultivars; however, it is difficult to select resistant cultivars because of the high level of genetic diversity of *A. citrulli*. Therefore, copper bactericides have widely been used to control BFB (4, 5). The long-term use of copper bactericides has led to the development of copper-resistant (Cu^r^) strains of *A. citrulli*, resulting in difficulties in disease control. Previous reports have shown that *A. citrulli* can be classified into two genetically distinct groups (6). In 2007, a draft genome sequence of *A. citrulli* AAC00-1, which belongs to group 2 (GenBank accession no. NC_008752), was reported. Compared to strain *A. citrulli* AAC00-1 and other *A. citrulli* strains, strain Tw6 was found to show an unexpectedly high capacity for tolerating copper sprays and antibiotics. In order to study the copper metabolism of *A. citrulli*, we announce here the determination of the draft genome of strain *A. citrulli* Tw6, which was isolated from a watermelon seedling displaying leaf scorch symptoms in Beijing, China.

The genome of this strain was sequenced with massively parallel sequencing (MPS) Illumina technology. Two DNA libraries were constructed, a paired-end library with an insert size of 300 to 400 bp, and a mate-pair library with an insert size of 5 kb. Using Velvet version 1.2.07, we assembled the genome into 25 scaffolds, with an average length of 203,224 bases. The largest one was 768,921 bases. Among the large scaffolds, the *N*_50_ contig size was 527,712 bases. Gene prediction was performed on the *A. citrulli* strain AAC00-1 genome assembly using GeneMarkS (http://topaz.gatech.edu/) (7). A whole-genome BLAST search (*E* value, ≤1e^-5^; minimal alignment length, ≥40%) was performed against 5 databases. They are the TREMBL (computer-annotated supplement to Swiss-Prot) (8), nonredundant (NR) protein (9), Swiss-Prot (10), Clusters of Orthologous Groups (COG) (11), and Gene Ontology (GO) (12) databases.

The genome contains a single circular chromosome of 5,080,614 bp, with a G+C content of 68.7%. The total coding genes are 4,491,540 bp, with a coding ratio of 88.41%. A total of 4,759 protein-coding genes were identified in the genome, with an average length of 943 bp. There are three rRNAs and 48 tRNAs.

### Nucleotide sequence accession numbers. 

This whole-genome shotgun project has been deposited at DDBJ/EMBL/GenBank under the accession no. JXDJ00000000. The version described in this paper is version JXDJ01000000.
